# Editorial: Experiments and Simulations: A *Pas de Deux* to Unravel Biological Function

**DOI:** 10.3389/fmolb.2021.799406

**Published:** 2021-11-29

**Authors:** Maya Topf, Edina Rosta, Gregory R. Bowman, Massimiliano Bonomi

**Affiliations:** ^1^ Center for Structural Systems Biology (CSSB), Leibniz-Institut für Experimentelle Virologie (HPI) and Universitätsklinikum Hamburg-Eppendorf (UKE), Hamburg, Germany; ^2^ Department of Chemistry, King’s College London, London, United Kingdom; ^3^ Department of Physics and Astronomy, University College London, London, United Kingdom; ^4^ Department of Biochemistry and Molecular Biophysics, Washington University School of Medicine, St Louis, MO, United States; ^5^ Structural Bioinformatics Unit, Department of Structural Biology and Chemistry, CNRS UMR 3528, Institut Pasteur, Paris, France; ^6^ USR3756 Centre de Bioinformatique, Biostatistique et Biologie Intégrative (C3BI), Paris, France

**Keywords:** modeling, molecular dynamic (MD), integrative approaches, functional dynamics, experimental-computational method, molecular simulation

Understanding the molecular mechanisms used by biological systems to perform their functions is often essential to rationally target associated diseases. In many cases, the determination of the three-dimensional structure of these systems provides precious insights. However, it is more often the interplay between structural and dynamical properties that determines the behavior of complex systems (Henzler-Wildman and Kern, 2007; Orozco, 2014). While both experimental and computational methods are invaluable tools to study protein structure and dynamics, limitations in each individual technique can hamper their predictive capabilities (Schneidman-Duhovny et al., 2014). On one hand, determining structural models solely from experimental data is challenging as these data often come from time and ensemble-averaged measurements over conformationally heterogeneous states, provide sparse and sometimes ambiguous information, and are always subject to random and systematic errors. On the other hand, structural models determined by computational approaches such as protein structure prediction methods and/or molecular dynamics (MD) are limited by the inaccuracies of the force fields used as well as by the challenge of exhaustively sampling the conformational landscape of complex systems (Bonomi et al., 2017). Combining experiments and simulations is therefore a successful strategy to overcome the limitations of the individual approaches and to accurately characterize the behavior of biological systems (Bottaro and Lindorff-Larsen, 2018; Rout and Sali, 2019).

The goal of this Research Topic is to present some representative examples of synergistic use of experimental and computational techniques aimed at accurately characterizing the structure, dynamics, and ultimately function of biological systems. This Research Topic of 14 articles explores different areas of experimental-computational integration: the use of computational approaches to assist the interpretation of existing experimental data or to predict the outcome of new measurements, the experimental validation of computational predictions, and the incorporation of experimental data to drive and/or refine molecular simulations. A wide spatial spectrum of systems will be covered, encompassing ordered and disordered peptides and proteins, small-molecules interacting with proteins, protein complexes, nucleic acids, and entire cells. The integration of molecular simulations with different types of experimental data will be illustrated, including cryo-electron microscopy (cryo-EM) and tomography, super-resolution microscopy, Nuclear Magnetic Resonance (NMR) spectroscopy, biochemical, and Small Angle X-ray Scattering (SAXS) data.

One of the areas of research in which computationally approaches, and particularly MD simulations, have traditionally been used to complement experimental measurements is the prediction and/or rationalization of the effect of mutations on the structure and dynamics of biological systems. Crnjar et al. use MD simulations to shed light into the effect of a single-point mutation in the outer lipid-facing helix (M4) of the 5-HT3A pentameric ligand-gated ion channels. The mutation of a tyrosine (Y441) in this area has been experimentally shown to inhibit the function of the receptor. The MD simulations reported in this paper reveal a network of interactions that connects Y441 to the ion channel hydrophobic gate on helix M2, thus rationalizing the effect of the mutation that has experimentally been observed. Ochoa et al. build a set of scoring matrices using structural observables extracted from MD simulations to predict the effect of single-point mutations on peptide binders to the Major Histocompatibility Complex (MHC) class II receptors. The method developed integrates sequence, structural and dynamical information and can be used to guide the design of novel peptide binders to the MHC class II receptors.

Another area in which molecular simulations can complement experiments is the characterization of conformations that are often difficult to observe directly, such as short-lived conformational states and disordered motifs. Nierzwicki and Palermo illustrate the case of the CRISPR-Cas9 genome editing machinery, of which the catalytically active structure has been predicted by MD simulations and subsequently validated by high-resolution cryo-EM data. This paper also provides an overview of computational approaches that can be used to refine both single-structure models and conformational ensembles given a cryo-EM map. Lambrughi et al. use enhanced-sampling MD simulations to study the Ubiquitin Interacting Motif (UIM), a conserved, highly-dynamic segment used by several multi-domain proteins to interact with ubiquitin. While existing X-ray data could not capture the structural heterogeneity of UIM, the MD ensembles revealed an equilibrium between ordered and disordered states, in agreement with NMR chemical shifts data.

Computational techniques have become over the years an invaluable tool in the drug discovery field (Brogi et al., 2020). In this Research Topic, De Felice et al. present an *in silico* approach named “Computational Profiling for GPCRs” that repurposes a GPCR-binding ligand for a different GPCR. The method is tested on 3 different GPCR receptors and validated using docking calculations and pharmacological data. Sgrignani and Cavalli use computational docking and molecular simulations to investigate the mode of binding of bromhexine to the transmembrane serine protease TMPRSS2, an enzyme involved in the activation of several coronaviruses, including SARS-CoV-2. Their analysis reveals the existence of an allosteric pocket involved in the binding of bromhexine to TMPRSS2 and in its inhibition. Finally,Prerovská et al. perform MD simulations to predict the structure of the complex formed by β-D-galactosyl Yariv reagent and oligo β-D-(1→3)-galactan and ultimately to shed light into the structural basis of arabinogalactan protein precipitation by Yariv.

Over the last decade, a novel class of methods that incorporate experimental information into molecular simulations has flourished. These so-called integrative or hybrid modeling approaches use experimental data to either guide or refine *a posteriori* (Rangan et al., 2018) molecular simulations in order to determine individual structural models or conformational ensembles consistent with the available information. These techniques are often based on 1) Bayesian frameworks to properly balance the information provided by different types of experiments with prior, physico-chemical knowledge of the system; and 2) the Maximum Entropy Principle to resolve the ambiguity of determining conformational distributions based on the knowledge of ensemble-averaged experimental observations (Ravera et al., 2016; Bonomi et al., 2017; Bottaro and Lindorff-Larsen, 2018; Cesari et al., 2018). In this Research Topics, six examples of this type of hybrid computational-experimental approaches are illustrated.

The papers by Ahmed et al., Spill et al., and Paissoni et al. present different ways to characterize conformational ensembles of dynamic systems by integrating MD simulations with SAXS data. Ahmed et al. use the refinement Bayesian/Maximum Entropy (BME) technique to determine structural ensembles of α-synuclein starting from MD simulations performed with different force fields. Spill et al. propose a Bayesian weighting approach for SAXS data coupled with a selection of an ensemble of minimal size to characterize the conformational heterogeneity of a tandem of domains from the protein PTPN4. Paissoni et al. perform an exhaustive analysis of the statistical precision of the metainference technique coupled with metadynamics using as test system the chignolin peptide. Voelz et al. present a review of the features, advantages over other integrative approaches, and shortcomings of their Bayesian Inference of Conformational Populations (BICePs) method. Gaalswyk et al. illustrate how their Modeling Employing Limited Data (MELD) approach can be used to determine protein structural ensembles using sparse NMR data combined with physical modeling. Finally, Vakili and Habeck introduce a Bayesian technique to address the problem of reconstructing, in tomography, a 3D structure from 2D views along unknown random directions.

The majority of applications of combined computational-experimental techniques presented in this Research Topic involve biological systems ranging in size, from small molecules to protein complexes. However, integrative approaches can in principle be applied to larger spatial scales, provided that appropriate experimental data are available. One illustrative example presented here is the work of Bianchi et al. In this paper, the authors combine single molecule super-resolution microscopy in cells with computational modeling to study how a small RNA (SgrS) regulates glucose-phosphate stress, or “sugar shock” in *E. coli.* Their stochastic simulations guided by the in cell experimental data enable the description of the cellular heterogeneity observed in the *E. coli* sugar shock response network.

To summarize, this Research Topic demonstrates how a synergistic use of experiments and simulations can be a powerful strategy to study structure, dynamics, and function of biological systems across a variety of spatial and temporal scales. These successes have been made possible by the continuous improvements in both experimental and computational techniques, the development of open-source software and web-servers for integrative studies (Rout and Sali, 2019), the dissemination of protocols in open-source notebooks and public repositories (The PLUMED Consortium, 2019), and the availability of raw data and structural models on public databases, such as the PDB-Dev^1^(Burley et al., 2017). There is a bright future ahead for integrative studies, especially these days, when a new class of methods based on artificial intelligence, such as DeepMind’s AlphaFold (Jumper et al., 2021) and RoseTTA fold (Baek et al., 2021), has joined the game and already taken it by storm, reaching accuracy in structure prediction comparable to experimental techniques.

## Footnotes

1
https://pdb-dev.wwpdb.org
